# Colorimetric Detection of Platinum (IV) Using 4-MethylSulfonylaniline-Modified Gold Nanoparticles in Lanthanum Carbonate API

**DOI:** 10.3390/s25113274

**Published:** 2025-05-23

**Authors:** Zhongqiu Li, Longwei Li, Xiaotian Yang, Mengtao Duan, Zhiwei Li, Shiguo Sun

**Affiliations:** 1 College of Chemical and Pharmaceutical Engineering, Hebei University of Science and Technology, 26 Yuxiang Road, Shijiazhuang 050018, China; 2 State Key Laboratory Breeding Base–Hebei Key Laboratory of Molecular Chemistry for Drug, 26 Yuxiang Road, Shijiazhuang 050018, China

**Keywords:** colorimetric detection, gold nanoparticles, 4-methylsulfonylaniline (4-MESA), Platinum (IV), active pharmaceutical ingredient (API)

## Abstract

The control of elemental impurities is a critical step in the preparation of lanthanum carbonate, with platinum being one such impurity. Residual platinum is typically non-therapeutic and must be strictly controlled to ensure both safety and product quality. This paper describes a colorimetric method for determining platinum (IV) in solutions based on the anti-aggregation of gold nanoparticles modified with 4-methylsulfonylaniline (4-MESA). The presence of Britton–Robinson buffer induces the aggregation of the 4-MESA-AuNPs nanoparticle probe. However, when platinum (IV) is introduced, it disrupts the aggregation of the 4-MESA-AuNPs, causing a color change in the solution. The absorbance at 524 nm showed a strong linear correlation in the concentration range of 1.00 × 10^−2^ μM to 5.00 × 10^2^ μM. Under optimal conditions, LOD and LOQ values of 10.00 × 10^−3^ μM and 3.03 × 10^−2^ μM, respectively, were observed. This method has been successfully applied to the determination of platinum (IV) in lanthanum carbonate API.

## 1. Introduction

Lanthanum carbonate (La_2_(CO_3_)_3_) chewable tablets, which were first developed by a British company (Shire Development), are used to treat hyperphosphatemia in patients with end-stage renal disease. Compared with calcium–phosphorus-binding drugs, lanthanum carbonate is more effective in the treatment of hyperphosphatemia and better tolerated [1,2]. An important step in the development of lanthanum carbonate preparations is the control of elemental impurities in the active pharmaceutical ingredient (API). The International Conference on Harmonization of Technical Requirements for Registration of Pharmaceuticals for Human Use (ICH): Guideline for Elemental Impurities Q3D(R1) [3] specifies the Permitted Daily Exposures (PDE) of 24 potentially risky elemental impurities in pharmaceutical products. Platinum (Pt) is also included. At present, there are few reports on the detection of elemental impurities in lanthanum carbonate API, and the complete detection method of Pt with potential risks has not been reported. Due to the potential toxicity of Pt [4], it is necessary to establish a complete, rapid and simple method for the determination of platinum.

Currently, several complex instrumental methods with low detection limits (LODs) have been used for the detection of Pt in different samples [5], such as atomic absorption spectrometry (AAS) [6,7,8,9,10], inductively coupled plasma mass spectrometry (ICP-MS) [11,12,13,14,15,16,17,18], flame atomic absorption spectrometry (FAAS) [19], neutron activation analysis (NAA) [20,21,22,23,24,25,26,27,28], and graphite furnace atomic absorption spectrometry (GFAAS) [29]. Due to the low content of Pt(IV), the presence of interfering components, and the complex composition of most practical samples, a complex pretreatment procedure (such as adsorption–coprecipitation, solvent extraction, ion exchange, flotation, etc.) is required [24,30,31,32,33,34,35,36]. In addition, the high price and operation cost have become common shortcomings of these methods [29].

Colorimetric determination of noble metal nanoparticles (NPs) as sensing elements has attracted great interest due to its unique advantages and the ability of visual detection [37]. Colorimetric sensors based on metal NPs have been widely used to monitor a variety of analytes depending on the analyte-induced reversible color transition between the dispersion-aggregation states of NPs [38,39,40,41]. To the best of our knowledge, gold nanoparticles (AuNPs) are one of the most commonly used nanomaterials. If 4-methylsulfonanilide (4-MESA) is modified on AuNPs’ surface, due to its high electronegativity [42,43], sulfonyl groups can form hydrogen bonds with substances in BR buffer, resulting in the aggregation of 4-MESA-AuNPs. However, the presence of Pt(IV) can prevent the formation of hydrogen bonds and lead to 4-MESA-AuNPs’ anti-aggregation. This principle can be used to detect Pt(IV).

Meanwhile, the analysis of heavy metal ions is usually carried out in solution, while Pt ions are mainly in the form of Pt(IV) [44,45,46]. Therefore, a visualized colorimetric sensing platform was proposed to detect Pt(IV) ions using chemical changes in ligands on the surface of modified AuNPs nanoparticle probes. In the detection system, 4-methylsulfonylaniline (4-MESA) was successfully modified to the surface of gold nanoparticles to form a 4-MESA-AuNPs nanoparticle probe solution. The method is accurate, sensitive, simple and feasible, and can be used for the determination of Pt in lanthanum carbonate API.

## 2. Experimental Procedure

### 2.1. Chemicals and Materials

4-methylsulfonylaniline (4-MESA) was purchased from Bidepharm (Shanghai, China). Britton–Robinson (BR) buffer solutions were purchased from Leagene (Beijing, China). HAuCl_4_·4H_2_O, Na_3_C_6_H_5_O_7_·2H_2_O, PtCl_4_, PtCl_2_ and other heavy metal ions were purchased from Beijing Chemical Company (Beijing, China). All reagents were of analytical grade and used without further purification. The solutions were prepared using high-purity water with a resistivity of 18 MΩ.cm. Lanthanum carbonate API was purchased from a production enterprise in Hebei, China (Lot number: 240501, 240603, 240706).

UV–vis absorption spectra were acquired on a UV-2550 spectrophotometer (Shimadzu, Japan), using 1 cm path length quartz cuvettes for measurements. The IR spectra were measured with an FT-IR spectrometer (Vertex 70, Bruker, Germany). Flame atomic absorption spectrometry (FAAS) experiments were carried out by using a PinAAcle 900T spectrometer (PerkinElmer, Waltham, MA, USA). A vortex mixer (IKA Lab Dancer, Shanghai, China) was purchased from Chuanxiang Biotechnology Co., Ltd. (Shanghai, China) The pH value of the solution was measured with a PB-10 pH meter (Sartorius, Göttingen, Germany). Transmission electron microscopy (TEM) images were acquired on a Hitachi HT7700 (Hitachi, Chiyoda, Japan). Dynamic light-scattering (DLS) data were measured by a DynaPro Nanostar (WYATT Technology Corporation, Goleta, CA, USA).

### 2.2. Preparation of 4-MESA-AuNPs

Gold nanoparticles with a concentration of 10.00 × 10^−3^ μM were synthesized according to the method described in the literature [47,48]. Briefly, 150.00 mL of a 1.00 × 10^3^ μM HAuCl_4_·4H_2_O solution was placed in a circular flask with a reflux device to heat until boiling. Then, 15.00 mL of a 3.88 × 10^4^ μM sodium citrate aqueous solution was quickly added with vigorous magnetic stirring. Under stirring and refluxing, the mixed solution was left to boil for another 15 min and the wine-red gold nanoparticle solution was formed. After cooling to room temperature, the solution was stored at 4 °C for further use.

4-MESA-AuNPs synthesis: 20.00 mL of the prepared gold nanoparticle solution was added to 80.00 mL of high-purity water for dilution. Then, 0.40 mL of the 1.00 × 10^4^ μM 4-MESA solution was added into the diluted gold nanoparticle solution. In order to ensure a complete interaction between 4-MESA and AuNPs, the 4-MESA-AuNPs nanoparticle probe solution was obtained after stirring for 2 h at room temperature.

### 2.3. Pt(IV) Sensing

The 0.1 g API sample was dissolved in 0.5 mL concentrated nitric acid and diluted to 25 mL with ultra-pure water. Subsequently, 2 mL of the diluent was added into ultra-pure water to obtain a 10 mL sample solution under test. The blank solution was prepared by the same method.

The BR buffer solution (pH = 8.0, 0.80 mL) and different concentrations of the Pt(IV) solution (0.20 mL) were added into the 4-MESA-AuNPs solution (2.00 mL), which contains the sample solution under test (1.00 mL). After reaction at room temperature for 1 min, the UV–vis spectra and colorimetric images of the mixed solution were recorded.

## 3. Results and Discussion

### 3.1. Characterization of 4-MESA-AuNPs and the Interaction Mechanism for Detecting Pt(IV)

To investigate the modification of 4-MESA-AuNPs, FT-IR spectroscopy was performed. The FT-IR spectra of pure 4-MESA and 4-MESA-AuNPs were shown in Figure S1. Compared with pure 4-MESA, the characteristic absorption peaks (3485 cm^−1^ and 3372 cm^−1^) of -NH_2_ in the FT-IR spectrum of 4-MESA-AuNPs disappeared, and the characteristic absorption peaks of -SO_2_CH_3_ appeared at 1242 cm^−1^ and 1062 cm^−1^, indicating that 4-MESA was successfully modified to the surface of AuNPs particles by the -NH_2_ group.

**Figure 1:**
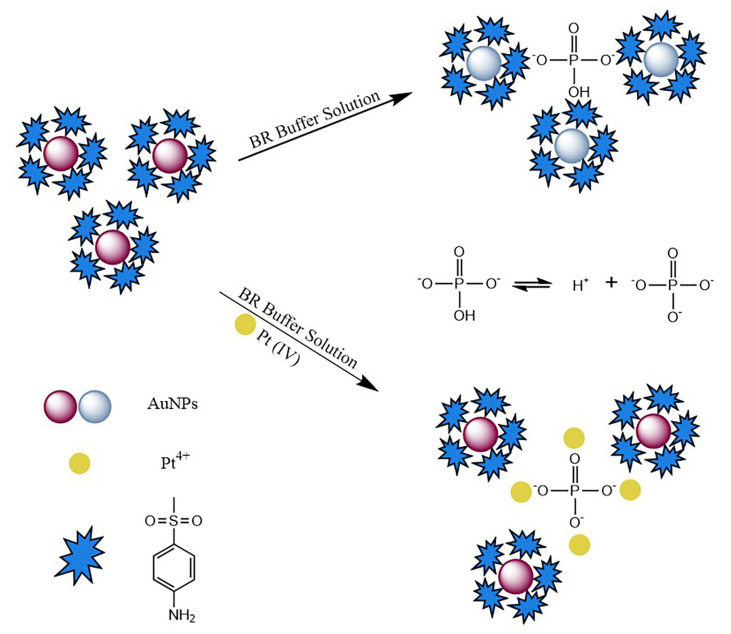
Schematic representation of the anti-aggregation of 4-MESA-AuNPs by Pt(IV).

The mechanism of interaction between 4-MESA-AuNPs nanoparticle probes and Pt(IV) is shown in Figure 1. It has been reported that BR buffer solutions of different pH values could affect the aggregation state of AuNPs [49,50]. The Britton–Robinson (BR) buffer solution is a mixture of 4.00 × 10^4^ μM phosphoric acid, acetic acid and boric acid (H_3_PO_4_-HAc-H_3_BO_3_). Different amounts of 2.00 ×10^5^ μM sodium hydroxide were added to form a buffer solution with a wide pH range (pH 1.8~11.9). In order to further explore the components, which led to the aggregation of AuNPs in the BR buffer solution, the effects of H_3_PO_4_, H_3_BO_3_, HAc and NaOH on the aggregation of AuNPs were investigated.

The results (Figure S2) prove that significant aggregation of the 4-MESA-AuNPs nanoparticle probe was only caused by the H_3_PO_4_ solution. Therefore, the possible reason for the aggregation of the 4-MESA-AuNPs nanoparticle probe under pH 8.0 was the presence of HPO_4_^2−^ in the BR buffer solution (the pK_a1_, pK_a2_ and pK_a3_ of H_3_PO_4_ were 2.12, 7.21, and 12.36). The -OH and -O^−^ of HPO_4_^2−^ could form hydrogen bonds with the -SO^2−^, -NH^−^ of the 4-MESA-AuNPs nanoparticle probe [51,52], resulting in the aggregation of the 4-MESA-AuNPs nanoparticle probe. Pt(IV) could coordinate with PO_4_^3−^, which destroyed the HPO_4_^2−^ dissociation equilibrium, promoting the ionization of HPO_4_^2−^, finally leading to the 4-MESA-AuNPs nanoparticle probe’s anti-aggregation. The degree of color change in the solution was related to the Pt(IV) concentration, which provided a visible or spectroscopic system for the qualitative and quantitative detection of Pt(IV).

**Figure 2:**
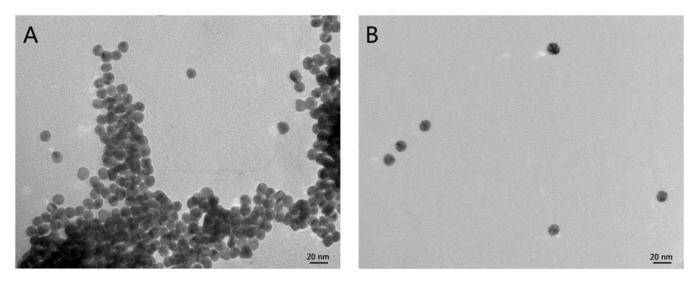
TEM images of 4-MESA-AuNPs: (**A**) aggregated 4-MESA-AuNPs in the absence of Pt(IV). (**B**) Dispersed 4-MESA-AuNPs in the presence of 100.00 μM Pt(IV).

**Figure 3:**
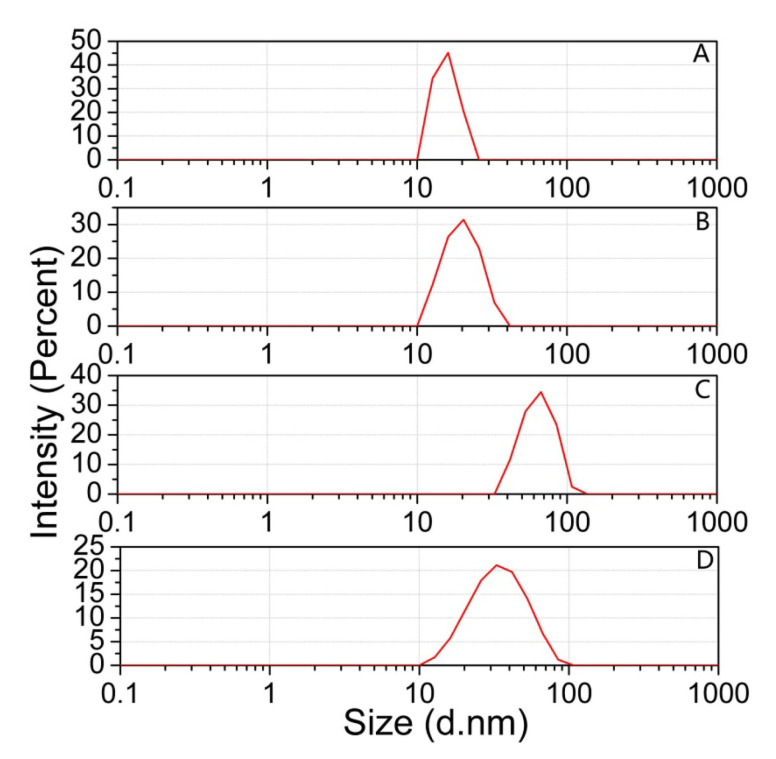
DLS measurements for (**A**) AuNPs, (**B**) 4-MESA-AuNPs, (**C**) 4-MESA-AuNPs + BR buffer solution, (**D**) 4-MESA-AuNPs + BR buffer solution + Pt(IV).

The detection mechanism was also verified by transmission electron microscopy (TEM). In the presence of BR buffer solutions, the aggregation of 4-MESA-AuNPs was observed (Figure 2A), confirming that the BR buffer solution could induced large-scale aggregation of 4-MESA-AuNPs. However, Figure 2B shows that 4-MESA-AuNPs were well dispersed in the BR buffer solution when 100.00 µM Pt(IV) was added. The hydrodynamic diameter of 4-MESA-AuNPs was measured by the DLS method at the same time and the size distribution of DLS is as shown in Figure 3. The average hydrodynamic diameter (d_h_) of unmodified gold nanoparticles estimated by DLS was 13.6 nm (Figure 3A). After 4-MESA was modified to the surface of AuNPs, the d_h_ was increased to 20.4 nm (Figure 3B), which indicated that 4-MESA had been successfully connected to AuNPs. When the BR buffer was mixed with the 4-MESA-AuNPs nanoparticle probe solution, the d_h_ was significantly increased to 66.9 nm (Figure 3C) by the aggregation of 4-MESA-AuNPs. In addition, 100.00 µM Pt(IV) was first mixed with the BR buffer, and then the 4-MESA-AuNPs nanoparticle probe solution was added. As shown in Figure 3D, the d_h_ was significantly reduced to 32.8 nm, indicating the anti-aggregation of 4-MESA-AuNPs. Based on the above experimental results, a simple and novel colorimetric detection method of Pt(IV) was proposed in this paper.

### 3.2. Optimization of Detection Conditions

In order to obtain a better detection response of Pt(IV), several experimental factors such as the pH value and the size of AuNPs were optimized before the application of this method.

#### 3.2.1. pH

**Figure 4:**
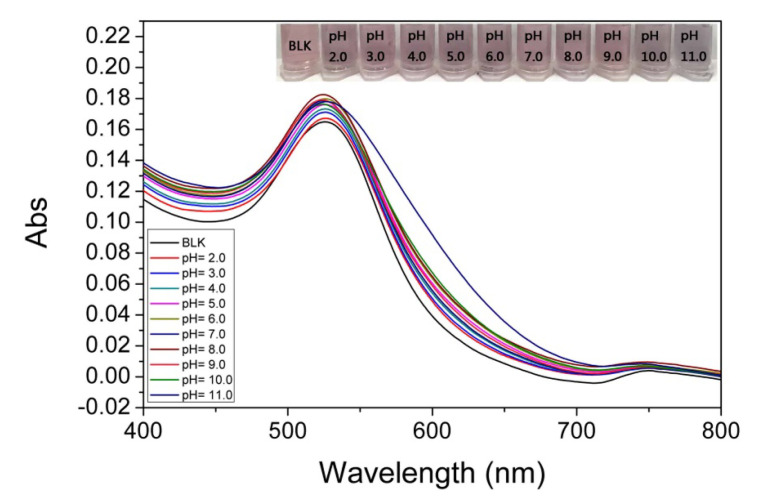
The colorimetric and UV–vis spectra results of 4-MESA-AuNPs solutions at the same concentration of Pt(IV) (100.00 μM) under different pH conditions.

Since pH plays an important role in the visual colorimetric detection of Pt(IV), the effect of pH on the detection system was investigated. As shown in Figure S3, the optimal pH of the 4-MESA-AuNPs probe was investigated by colorimetric selectivity of Pt(IV) under different pH conditions (pH = 5.0~10.0). Hg^2+^, Fe^3+^, Fe^2+^, Mn^2+^, Pb^2+^, Zn^2+^, Ni^2+^, Co^2+^, Cu^2+^, Mg^2+^, Cd^2+^, Cr^3+^, Pt^4+^, Pt^2+^, K^+^, Na^+^, and Li^+^ (100.00 μM) were added into the mixture solution of 4-MESA-AuNPs and different pH BR buffers. As can be seen from Figure S3, the best colorimetric selectivity of 4-MESA-AuNPs nanoparticle probe for Pt(IV) was obtained at pH = 8.0. At the same time, the UV–vis spectra of the 4-MESA-AuNPs nanoparticle probe solution with same concentration of Pt(IV) (100.00 μM) under different pH values (pH = 2.0~11.0) were also measured. The results (Figure 4) showed that UV–vis absorption of the 4-MESA-AuNPs nanoparticle probe solution at pH = 8.0 was more sensitive than that at other pH values. Based on the above results, pH 8.0 was chosen as the best pH value for the detection of Pt(IV) in the subsequent experiments.

#### 3.2.2. The Size of AuNPs

**Figure 5:**
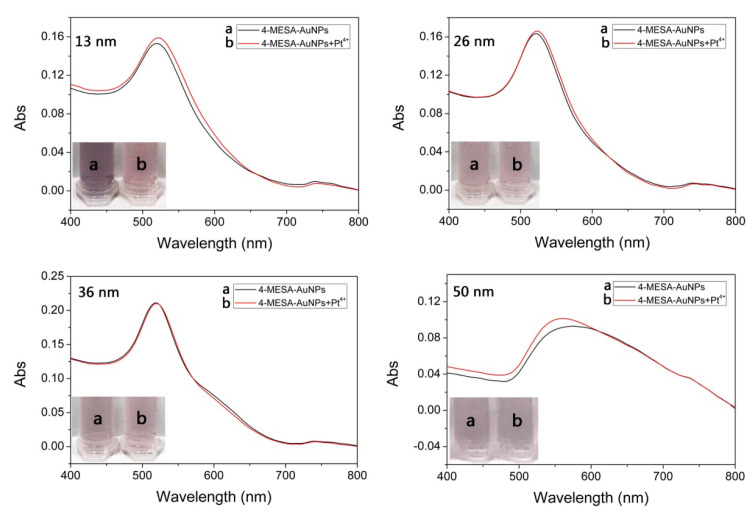
Effect of particle size on the aggregation of 4-MESA-AuNPs in the presence of Pt(IV) (100.00 μM).

The sensitivity of the colorimetric method could be improved with the appropriate particle size of AuNPs [53,54]. AuNPs with different sizes (13 nm, 26 nm, 36 nm and 50 nm) could be synthesized from different proportions of trisodium citrate and chloroauric acid according to the literature [55]. Therefore, AuNPs with different sizes were modified under the same concentration of 4-MESA. Then, the BR buffer solution (pH = 8.0) and Pt(IV) with the same concentration were mixed and added into the 4-MESA-AuNPs solution. As shown in Figure 5 and Table S1, the color change in the 4-MESA-AuNPs solution that was synthesized from the size of AuNPs at 13 nm was most obvious. The reason might be that the smaller the size of the AuNPs, the more 4-MESA-AuNPs were combined with a single HPO_4_^2−^, which caused the higher aggregation of the 4-MESA-AuNPs probe. When the same concentration of Pt(IV) was added, PO_4_^3−^ was consumed by Pt(IV), resulting in increased dissociation of HPO_4_^2−^ and anti-aggregation of 4-MESA-AuNPs. Therefore, a diameter of 13 nm was selected as the optimum size of AuNPs.

### 3.3. Detection of Pt(IV) Using 4-MESA-AuNPs

**Figure 6:**
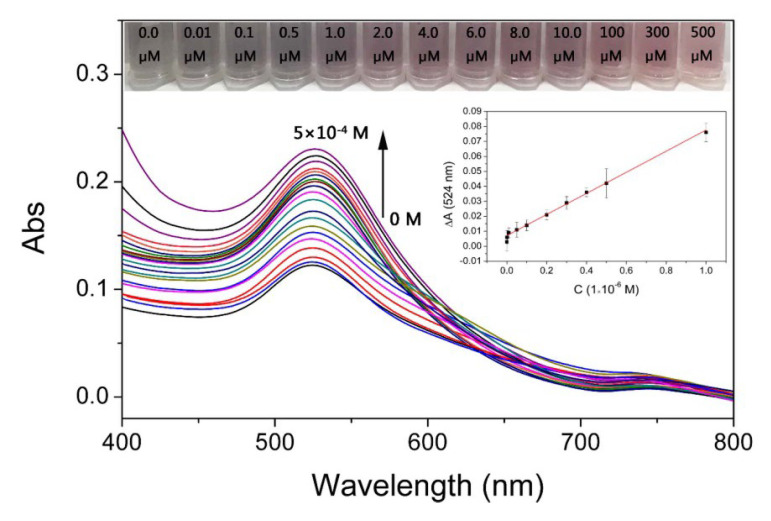
The UV–vis spectra of 4-MESA-AuNPs solutions with various concentrations of Pt(IV) ranging from 0.00 μM to 5.00 × 10^2^ μM. (From bottom to top, the concentrations of Pt(IV) were 0.00 μM, 0.01 μM, 0.10 μM, 0.20 μM, 0.30 μM, 0.40 μM, 0.50 μM, 0.60 μM, 0.70 μM, 0.80 μM, 0.90 μM, 1.00 μM, 2.00 μM, 4.00 μM, 6.00 μM, 8.00 μM, 10.00 μM, 100.00 μM, 200.00 μM, 300.00 μM, 400.00 μM, 500.00 μM.)

In order to verify the good performance of the sensor for detecting Pt(IV), a digital camera and ultraviolet–visible spectrophotometer were used to record the changes in color and UV–vis spectra of 4-MESA-AuNPs in the presence of Pt(IV) at different concentrations. The BR buffer solution (pH = 8.0, 0.80 mL) and different concentrations of Pt(IV) solution (0.20 mL) were added into the 4-MESA-AuNPs solution (2.00 mL). Under the optimum conditions (pH is 8.0 and reaction time is 1 min), the colorimetric assay was performed using 4-MESA-AuNPs to detect a series of Pt(IV) solutions with concentrations ranging from 0.00 μM to 5.00 × 10^−1^ μM (Figure 6).

As can be seen from Figure 6, when the concentration of Pt(IV) was 1.00 µM, the color change in the 4-MESA-AuNPs solution was significant, which achieved the effect of visual colorimetric detection. From the UV–vis spectra in Figure 6, the presence of Pt(IV) caused the increase in the peak at 524 nm, and the absorption at 524 nm was gradually enhanced with the increase in Pt(IV) concentration, indicating that the anti-aggregation degree of 4-MESA-AuNPs was related to the concentration of Pt(IV).

With the concentration of Pt(IV) ranging from 1.00 × 10^−2^ μM to 5.00 × 10^2^ μM, a good linear relationship between the concentration of Pt(IV) and ΔA_524_ (ΔA_524_ = the absorption value of 4-MESA-AuNPs solution containing Pt(IV) at 524 nm minus the absorption value of the 4-MESA-AuNPs solution without Pt(IV) at 524 nm) was obtained. The linear relationship equation was ΔA_524_ = 0.00668 + 0.07035 × C(1.00 μM) (R^2^ = 0.9946), (Inset of Figure 6). The LOD and LOQ of the sensor were calculated as 10.00 × 10^−3^ μM and 3.03 × 10^−2^ μM, respectively. Compared with the detection methods reported in the literature (Table 1), the developed system was the most simple and sensitive.

### 3.4. Selectivity of 4-MESA-AuNPs

**Figure 7:**
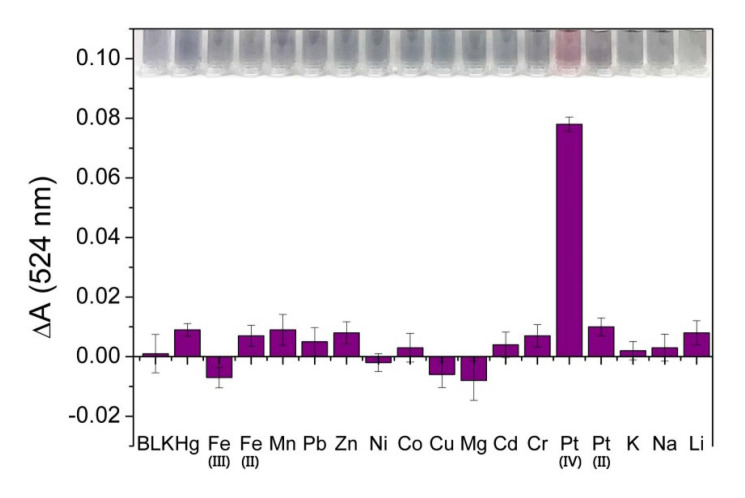
The visual and UV-vis spectra results of 4-MESA-AuNPs to various metal ions (10.00 µM) in aqueous phase.

In order to study the selectivity of this method, Hg^2+^, Fe^3+^, Fe^2+^, Mn^2+^, Pb^2+^, Zn^2+^, Ni^2+^, Co^2+^, Cu^2+^, Mg^2+^, Cr^3+^, Cd^2+^, Pt^2+^, K^+^, Na^+^ and Li^+^ with the same concentrations (1.00 × 10^2^ μM) were parallelly mixed with the BR buffer solution (pH = 8.0) and added to the 4-MESA-AuNPs solution. The visualized colorimetric detection image and UV–vis absorption spectra are shown in Figure 7. The value of ΔA_524_ was obviously increased only in the presence of Pt(IV), which means that the most significant anti-aggregation of 4-MESA-AuNPs was caused by Pt(IV). The results indicate that the presence of other ions had little interference with the detection of Pt(IV) due to the combination of Pt(IV) and PO_4_^3−^ being easier than that of other metal ions in the solution with a pH condition of 8.0, and the dissociation degree of HPO_4_^2−^ was the highest. Therefore, it was suggested that this colorimetric detection method had good selectivity.

### 3.5. Application to Lanthanum Carbonate API

To evaluate the applicability of the proposed sensing strategy in actual sample analysis, a standard addition method was used to detect lanthanum carbonate API samples. A series of standard solutions of Pt(IV) (10.00, 50.00, and 100.00 μM) were added into samples under test, and 4-MESA-AuNPs based on colorimetry were used to detect Pt(IV). As shown in Table 2, the recovery rates of the samples were found to be in the range of 96.60~108.60%, and the relative standard deviation (RSD) was 1.10~3.37%, which indicated that the 4-MESA-AuNPs probe was feasible and practical for the determination of Pt(IV) in lanthanum carbonate API.

The reference values of PDE for elemental impurities in oral preparations specified in ICH Q3D are shown in Table 3. According to the maximum possible daily intake of 3.75 g in the instruction manual of Fosrenol, the results of Pt(IV) in the three batches of lanthanum carbonate APIs did not exceed the PDE value.

## 4. Conclusions

In this paper, a simple, sensitive colorimetric detection method for Pt(IV) at room temperature was developed by using the anti-aggregation of 4-MESA-AuNPs. Under optimal conditions, this method showed a good linearity from 1.00 × 10^−2^ μM to 5.00 × 10^2^ μM with a coefficient of determination (R^2^) of 0.9946 and the detection limit was as low as 10.00 × 10^−3^ μM. Moreover, the proposed method was also successfully applied to the detection of Pt(IV) in lanthanum carbonate API. To sum up, a visual detection method of Pt(IV) is proposed for the first time. This colorimetric detection method for Pt(IV) not only has the advantages of being simple, sensitive and low-cost, but also expands new ideas for the detection methods of elemental impurities in API. The related concepts adopted in the establishment of the method are also applicable to the analysis of elemental impurities in other drugs.

## Figures and Tables

**Table 1:** Comparison of the detection methods of Pt(IV).

Methods	Detection	Linear Range (μM)	LOD (μM)	LOQ (μM)	Detection Time (min)	Reference
AAS	GFAAS	1.50 × 10^3^ to 3.55 × 10^4^	1.50	4.55	60	[29]
AAS	GFAAS	-	70.00	2.12 × 10^2^	180	[56]
AAS	GFAAS	-	4.80 × 10^3^	1.45 × 10^4^	3600	[57]
AAS	FI-column-GFAAS	-	5.05 × 10^3^	1.53 × 10^4^	3600	[58]
Fluorescent	CQDs	6.00 to 96.00	6.57 × 10^−1^	0.24	Not given	[59]
Colorimetry	AuNPs, 4-(Methylsulfonyl)aniline	1.00 × 10^−2^ to 5.00 × 10^2^	10.00 × 10^−3^	3.03 × 10^−2^	2	This work

LOD (limit of detection) = 3.3 × SD/slop, and LOQ (limit of quantification) = 10 × SD/slop.

**Table 2:** Analytical results for detection of Pt(IV) in lanthanum carbonate API.

Lot No.	Sample No.	Added (µM)	Found (µM)	Recovery (%)	RSD (%)
240501	1	10.00	9.68 ± 0.02	96.80	1.10
	2	50.00	48.30 ± 0.04	96.60	1.17
	3	100.00	99.00 ± 0.03	99.00	3.37
240603	1	10.00	9.95 ± 0.06	99.50	3.36
	2	50.00	51.20 ± 0.04	102.40	1.78
	3	100.00	99.56 ± 0.07	99.56	2.47
240706	1	10.00	10.86 ± 0.02	108.6	1.50
	2	50.00	49.98 ± 0.06	99.96	2.20
	3	100.00	99.97 ± 0.02	99.97	1.68

**Table 3:** Safety evaluation of test results.

Element	ICH PDE (μg·d^−1^)	Control Threshold (μg·d^−1^)	Maximum Possible Daily Intake (μg·d^−1^)			Conclusion
			240501	240603	240706	
Pt	100	30	˂0.18	˂0.20	˂0.18	Below limit

## Data Availability

Data are contained within the article.

## Supplementary Materials

The supporting information (Figures S1–S3 and Table S1) can be downloaded at: https://www.mdpi.com/article/10.3390/s25113274/s1.
