# Supplementary material for: Colorimetric Detection of Platinum (IV) Using 4-MethylSulfonylaniline-Modified Gold Nanoparticles in Lanthanum Carbonate API
Source: Sensors (Basel). 2025 May 23;25(11):3274. doi: 10.3390/s25113274 (PMC12157221; doi:10.3390/s25113274)
Supplement: Supplementary file 1 [file sensors-25-03274-s001.zip › sensors-3415600-supplementary.pdf]

Supporting Information

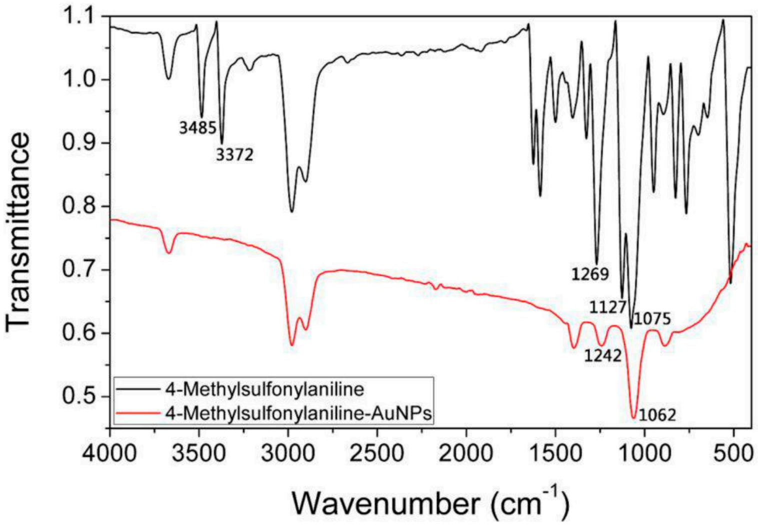

Figure S1 Infrared spectrograms of 4-MESA and 4-MESA-AuNPs.

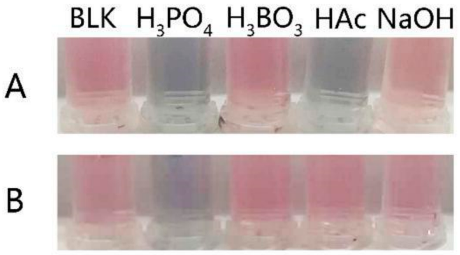

Figure S2 Colorimetric images of AuNPs (A) and 4- MESA-AuNPs (B) reacting with H_3PO_4 (0.04M), HAc (0.04M), H_3BO_3 (0.04M), and NaOH (0.20M), respectively.

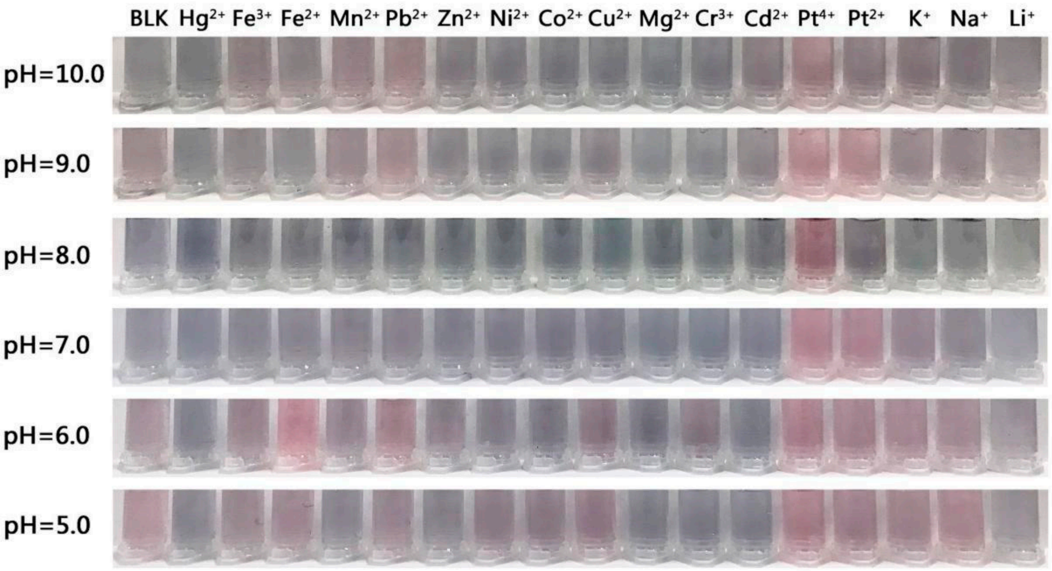

Figure S3 The colorimetric results of 4-MESA-AuNPs to various metal ions (1.00×10^{-4} M) under different pH conditions.

Table S1 Hydrodynamic diameter of nanoparticles' measurement using DLS.

	DLS d_h (nm)			
AuNPs (nm)	13	26	36	50
4-MESA-AuNPs	20.4	32.9	43.3	58.4
4-MESA-AuNPs + BR	66.9	80.6	90.5	104.6
4-MESA-AuNPs + BR + Pt(IV)	32.8	78.8	89.9	102.3
